# Integrating Biological and Radiological Data in a Structured Repository: a Data Model Applied to the COSMOS Case Study

**DOI:** 10.1007/s10278-022-00615-w

**Published:** 2022-03-16

**Authors:** Noemi Garau, Alessandro Orro, Paul Summers, Lorenza De Maria, Raffaella Bertolotti, Danny Bassis, Marta Minotti, Elvio De Fiori, Guido Baroni, Chiara Paganelli, Cristiano Rampinelli

**Affiliations:** 1 Dipartimento Di Elettronica, Informazione E Bioingegneria, Politecnico Di Milano, Milano, Italy; 2 Division of Radiology, IEO, European Institute of Oncology IRCCS, Milan, Italy; 3 Institute for Biomedical Technologies, National Research Council (ITB-CNR), Segrate, Italy; 4 Division of Data Management, IEO European Institute of Oncology IRCCS, Milan, Italy; 5 School of Medicine, University of Milan, Milan, Italy; 6 Bioengineering Unit, CNAO Foundation, Pavia, Italy

**Keywords:** Structured reporting, Standardization, Lung cancer screening, Radiology workflow

## Abstract

Integrating the information coming from biological samples with digital data, such as medical images, has gained prominence with the advent of precision medicine. Research in this field faces an ever-increasing amount of data to manage and, as a consequence, the need to structure these data in a functional and standardized fashion to promote and facilitate cooperation among institutions. Inspired by the Minimum Information About BIobank data Sharing (MIABIS), we propose an extended data model which aims to standardize data collections where both biological and digital samples are involved. In the proposed model, strong emphasis is given to the cause-effect relationships among factors as these are frequently encountered in clinical workflows. To test the data model in a realistic context, we consider the Continuous Observation of SMOking Subjects (COSMOS) dataset as case study, consisting of 10 consecutive years of lung cancer screening and follow-up on more than 5000 subjects. The structure of the COSMOS database, implemented to facilitate the process of data retrieval, is therefore presented along with a description of data that we hope to share in a public repository for lung cancer screening research.

**Supplementary Information:**

The online version contains supplementary material available at 10.1007/s10278-022-00615-w.

## Introduction

The term “biomarker” [1, 2] has become a central concept in the era of precision medicine, with a broad definition encompassing information from molecular, histologic or physiologic characteristics, as well as quantitative parameters extracted from medical imaging. In large part however, these fields have evolved independently leading to heterogeneity of data and isolation of datasets. Integrating so-called -omics data across types and sources in order to facilitate clinical trials [3, 4] and support discovery and validation of findings, argues strongly for the establishment of structured repositories.

Progress has been made in the area of non-digital biological samples through initiatives such as the BBMRI-ERIC (“Biobanking and BioMolecular Re-sources Research Infrastructure-European Research Infra- structure Consortium”) dedicated to support European biobanks [5]. BBMRI-ERIC have created guidelines to facilitate biological data exchange among institutions, which have resulted in the Minimum Information About BIobank data Sharing (MIABIS) [6]. In its most recent extension, MIABIS generalizes the concept of biobanks to provide recommendations on how to describe a wider range of datasets in terms of the nature of the samples, the sample donors, scientific research on samples, and the associated data [7]. These recommendations cover a wide range of fields, distinguishing MIABIS from other more focus-specific initiatives such as the International Classification of Disease for Oncology [8], which proposed an ontology of oncologic pathologies, or the Systematized Nomenclature of Medicine Clinical Terms (SNOMED CT) [9], which is aimed at standardizing clinical terms to report findings, symptoms, diagnoses and procedures. These initiatives tend to focus on a lower-level description of the data with respect to the general view provided by the MIABIS data model. In addition, MIABIS is moving to accommodate the concept of digital biological imaging samples and data-driven biobanks, according to the need for data integration in structured repositories.

In parallel with the growing use of biological and clinical imaging is their quantitative analysis via computer assisted diagnosis (CAD) systems exploiting radiomics [10, 11] and artificial intelligence (AI) [12] approaches to support time-consuming and error-prone research and clinical procedures [13, 14]. A critical component in developing applications based on these new technologies is the availability of labelled data in sufficiently large cohorts to identify and validate correlations between radiological imaging and the pathological substrate or patient prognosis [15, 16]. In this context, a small number of public imaging archives have been established to allow sharing imaging data among institutions [17], that are often used to externally validate derived imaging biomarkers.

Data sharing, therefore, has a fundamental role in advancing both research and clinical analyses [18–20]. Apart from some indications reported by Castro et al. [16] and Finke et al. [21] however, there are limited guidelines in the literature [21] on documenting imaging acquisitions and associating images to other clinical information. Thus, the possible definition of queries remains limited, and the transition between datasets is not straightforward. These limits on sharing data are even more evident when dealing with large datasets where thousands of images are collected, such as the case of lung cancer screening studies [22]. With lung cancer being one of the leading causes of cancer-related death, multiple institutions have carried out screening programs [23–25] based on low-dose Computerized Tomography (LDCT) to understand the potential of the prevention program, and subsequently define strategies for pulmonary nodule management. Among the different lung cancer screening programs, the Continuous observation of SMOking Subjects (COSMOS) study conducted at the European Institute of Oncology (IEO, Milano, Italy) is one of the largest for number of patients involved (> 5000) and length of follow-up period (10 years)[26].

In this work, we propose an extension of the MIABIS to digital imaging samples with the aim to integrate heterogeneous data, such as biological samples and digital imaging samples, in a structured database in favour of multicentric studies in precision medicine. To demonstrate the feasibility of this extension, we adopted the COSMOS lung cancer screening program as a case study, where radiological imaging and biological findings were collected. The structured database designed, here connects longitudinal LDCT imaging data to lesion-specific radiological features as well as to pathological results coming from non-digital biological samples. Following a general overview of the COSMOS database, the implementation of the proposed extension of MIABIS is put forward as a model for similar image-based datasets to foster research studies on lung cancer management.

## Materials and Methods

The proposed extension of MIABIS data model to digital imaging samples was defined through clarifications on pre-existing MIABIS relationships but especially reducing the minimum list of attributes needed to describe a *Sample* component. This allows for a generalizable *Sample* component to non-digital biological samples and digital imaging samples of different nature (e.g., biological imaging samples and/or radiological imaging samples). *Implication* and *Sub-event* components are also introduced as solutions to connect heterogeneous data. The extended data model was then applied to the COSMOS study.

### MIABIS Extension to Digital Imaging Samples

The extension of MIABIS proposed in this section retains the definitions of *Sample, Sample Donor* and *Event* given by Eklund N. (2020), which are the fundamental components considered by MIABIS:

- *Sample Donor* is a person who is a source of either a biological material or a digital representation of a biological entity such as an image.
- A *Sample* is a portion or quantity of biological material that is collected from a *Sample Donor*, or which is a digital representation of a biological entity of the *Sample Donor*, such as an image.
- An *Event* is something that happens in a given place and time and is related to the *Sample* and/or *Sample Donor*.

The link between *Sample* and *Event* is defined through an explicit connection represented as:

- a $\mathrm{many}-\mathrm{zero}$ (*Sample – Event*) relation or,
- a $\mathrm{one}-\mathrm{zero}$ (*Sample -Event*) relation.

since multiple samples can be acquired through the same event and, consistent with the above definition of *Event* (“and/or”), the existence of an event can also be independent from a sample and a *Sample* may or may not be associated with an *Event.*

**Fig. 1:**
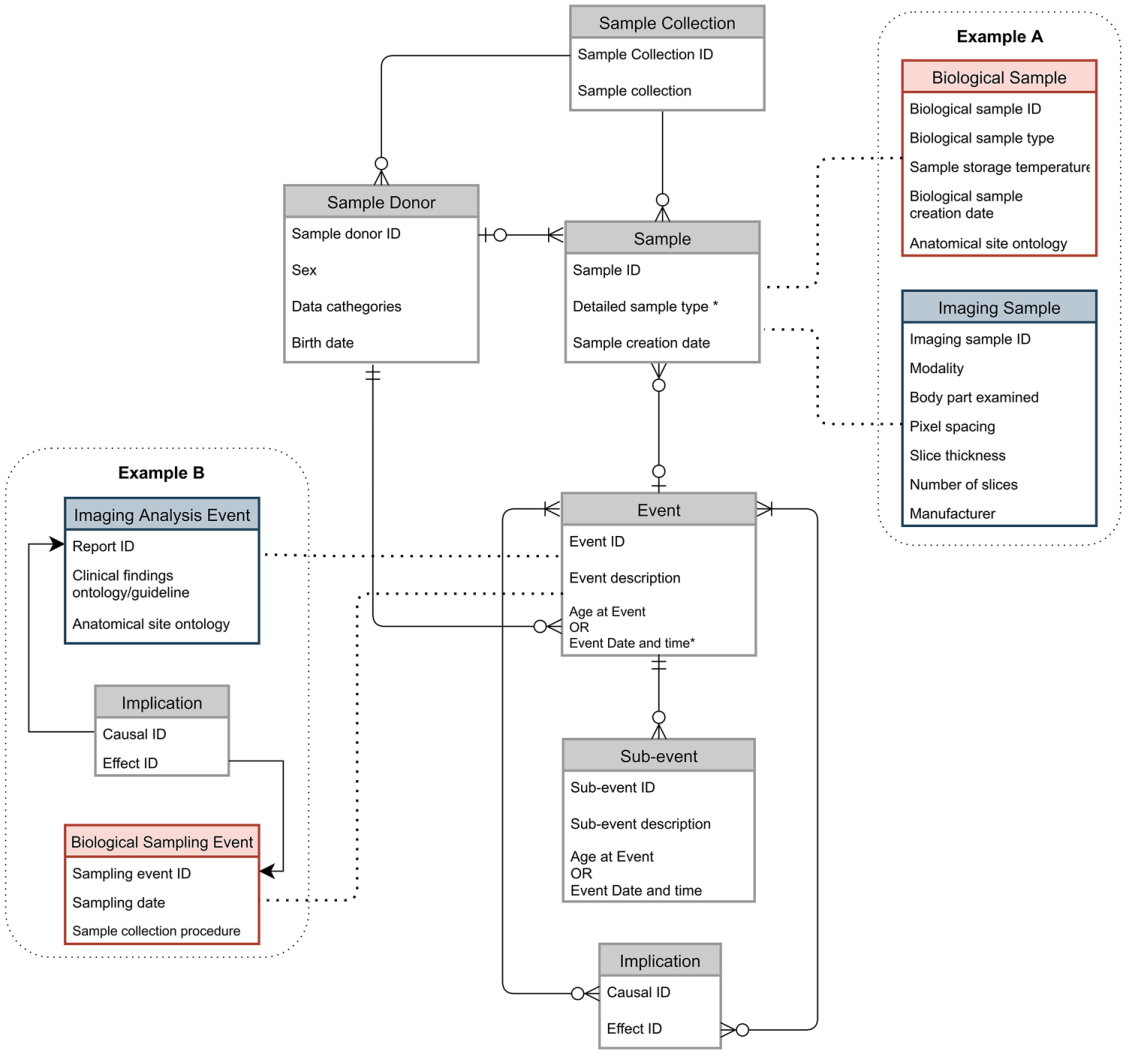
Illustration of the extended MIABIS data model components and relation structure. As per the diagram defined by Eklund et al. (2020), relations between *Sample*, *Sample Donor* and *Event* are maintained, while the *Implication* and *Sub-event* components are here introduced. In example A, the reduction of core characteristics of the samples permits generalization of sample types, with the type specific characteristics being deferred to the sub-collections of the biobank. Apart from relying implicitly on the chronology of events, the implication component (Example B) captures the causal relationship between events. Symbols used for connecting components represent the cardinality of the relation; refer to Supplementary Materials (section C) for more details about their meaning

With respect to the original attributes associated to MIABIS data model components, a change introduced for the extension of the MIABIS data model, was to reduce the number of attributes of the *Sample* component, making it generalizable to both non-digital biological and digital imaging samples. To make feasible the inclusion of radiological imaging samples, we removed attributes that were relevant exclusively to biological samples (e.g. the *storage temperature*). Such modality-specific attributes of samples can be contained in characteristics of specific sample variants (Fig. 1).

Two components were also introduced in the extension, specifically the *Implication* and the *Sub-event* components, defined respectively as follows:

- An *Implication* is a supportive component, which connects *Events* that are linked by a cause-effect relationship; this component indicates the presence of two *Events* where the first has a causal function towards the second *Event*, i.e. the *Event* is an effect.
- A *Sub-event* is an event that can be considered as part or product of a parent *Event*.

As both, *Implications* and *Sub-events,* depend on the *Event* component, the proposed extension of MIABIS impacts most on the place of events in the data model. Specifically, *Events* and *Implications* can be linked through.

- a $\mathrm{many}-\mathrm{one}$ (*Event-Implication*) relation or,
- a $\mathrm{many}-\mathrm{zero}$ (*Event-Implication*) relation

since the presence of an *Implication* has to assume the existence of at least one causal and one effect *Event,* but an *Event* can also not be associated to an *Implication* (an Event has not necessarily the property of being a cause or an effect).

As a generic example of the extended MIABIS data model, Fig. 1 illustrates two *Sample* components that are members of a biological (red) and digital imaging (blue) collection (Example A), respectively. Similarly, a connection between two Events mediated by an *Implication* is shown (Example B subfigure), wherein the *Imaging Analysis Event* (e.g. suspicion of disease based on the evidence of a radiological digital image) represents the cause that motivates the *Biological Sample Event* as effect (e.g. a more advanced clinical investigation).

### COSMOS as Case Study

#### The COSMOS Study Protocol

Between 2004 and 2015 a total of 5206 patients were recruited for the Continuous Observation of SMOking Subjects (COSMOS) non-randomized Lung Cancer Screening Study carried out at the European Institute of Oncology (IEO). The local ethical committee approved the study, and all participants provided signed informed consent for the study. The volunteers underwent annual LDCT scan for ten years [25, 26]. Eligibility criteria were to be at high risk of lung cancer occurrence: current or former heavy smokers (> = 20 pack years), and to be at least 50 years of age. The management of indeterminate pulmonary nodules through non-invasive procedures was one of the major aims of the protocol tested during the COSMOS study. Specifically, pulmonary nodules in the range 5–8 mm were scheduled to repeat LDCT after 3 or 6 months, whereas lesions above 8 mm or fast-growing lesions (i.e. volume doubling time between 30 and 400 days) were scheduled for a combined CT- positron emission tomography (CT-PET). Lesions with high probability of malignancy according to PET or growth rate, were further analysed through biopsies or other invasive procedures which, according to the adopted protocol, were minimized in favour of further diagnostic imaging acquisitions. In this study, only the LDCT images were incorporated as imaging samples into the structured database along with events of biological sampling, because the latter were collected without any form of digitalization.

#### Description of Deidentification and Annotation Collection Procedure

Prior to creation of the imaging repository with the extended MIABIS structure, radiological LDCT data were subjected to “pseudonymization” according to the European general data protection regulation (GDPR) [27], to facilitate research use within our institution and in collaboration with other institutions [28]. The LDCT scans were transferred from the hospital PACS (Picture Archiving and Communication System), into an Orthanc PACS [29]. Inside the Orthanc PACS a de-identification procedure was applied creating for each image an instance pseudonymized copy. Specifically, the institutional patient ID as well as the patient’s name were replaced with a pseudonym specific to the subject; the date of birth was indirectly masked including a patient age range while private tags were removed. Screenshots, dose reports and other DICOM files containing "burned-in" data were removed from the dataset. The entire procedure was managed through a python script.

The pseudonymized images were exported to patient-specific folders separated into subfolders for each study date, and sub-subfolders specific for each DICOM series. Each series was therefore associated with a specific path, which was then recorded into the structured database, along with the related DICOM fields as an instance of the table related to digital sample collection.

As the segmentation of the lesion is necessary for many analyses and for validation of CAD systems, we considered the availability and storage of labelled data fundamental in the creation of the repository. Once exams exportation was finalized, for annotation of lung nodules, a customized GUI was used for semi-automatic lesion segmentation with the possibility of a manual adjustment of the contour [30]. Each segmentation was stored as a DICOM structured reporting (DICOM-SR) file which allows encoding measurements referred to in the region of interest as well as information related to the DICOM series used to create the contours, where the latter are defined through a DICOM segmentation object (DICOM-SEG) linked to the DICOM-SR [31]. The DICOM-SR files were created taking advantage of an open source library implemented to store post-processed information of lesions (i.e. texture information) as DICOM tags [32]. As for the DICOM series, tags of the DICOM-SR were mapped in the structured database as attributes of the *Sub-event*.

#### COSMOS Structured Database Architecture

**Fig. 2:**
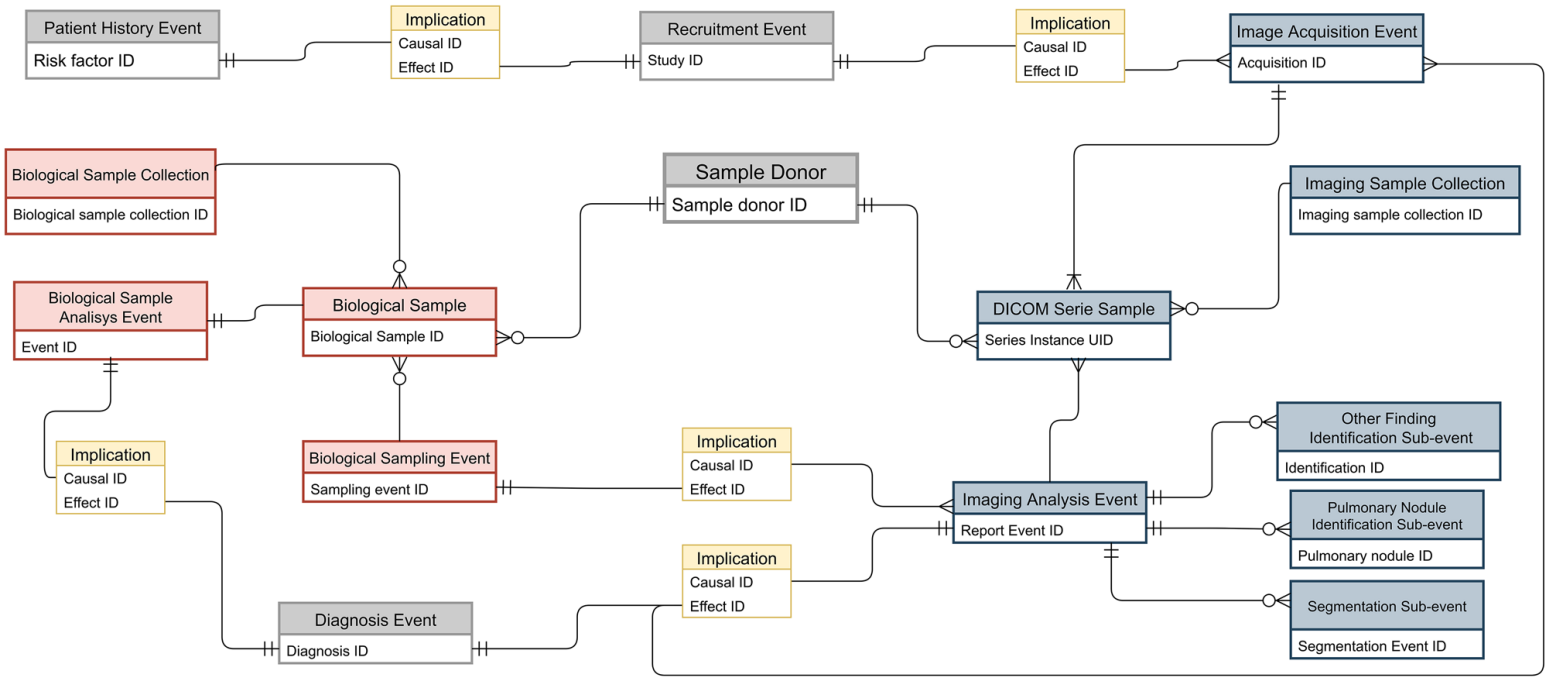
Simplified Schematic Relational Diagram describing the main components identified in the COSMOS database along with the main relationships among them. Relationships with the *Sample Donor* are explicitly shown only for the *Biological Sample* and the *DICOM Series Sample,* but the same connections exist between *Sample Donor* and the various *Events* contained in the diagram. Symbols used for connecting components represent the cardinality of the relation for the case study considered; refer to Supplementary Materials (section C) for more details about their meaning

The structured database for the COSMOS data was defined following the proposed extension of the MIABIS data model reported in “MIABIS Extension to Digital Imaging Samples”. The simplified relational diagram shown in Fig. 2 gives an overview of the relations among *Sample Donors*, *Samples* and *Events* for the COSMOS case study.

Two sample collections were defined: a *Biological Sample Collection,* which includes non-digital biological samples acquired through invasive procedures, and an *Imaging Sample Collection* consisting of LDCT scans. The two collections share the *Sample Donor* (Table S9, Supplementary Material, section A) from the patients recruited for the COSMOS study.

In defining the samples in the *Imaging Sample Collection*, we adopted the *DICOM Series Sample*, built upon the DICOM format [33], which is the international standard to transmit, store, retrieve, print, process, and display medical imaging information; as such, most of the well-defined DICOM fields were considered as attributes to define the imaging sample component (Table S1, supplementary materials, section A).

For the COSMOS case study, two events were directly connected to the *DICOM Series Sample*:

- the *Image Acquisition Event* (Table S2, supplementary materials, section A), which holds the information needed to contextualize the radiological imaging sample into the medical history of the *Sample Donor,* e.g. whether the acquisition is the first (“baseline”) of the longitudinal series or if it is located at a subsequent time-point (“routine or monitoring follow-up”).
- the *Imaging Analysis Event*, which represents the products derived from the radiological imaging sample, consisting of clinical findings or reports as well as digital data obtained by processing the imaging sample. Products of the same category were grouped into the same *Sub-event* of the *Imaging Analysis Event.* Specifically, the following three *Sub-events* were defined as products of the *Imaging Analysis Event:*
  - the *Pulmonary Nodule Identification Sub-event* (Table S3, supplementary materials, section A), to collect radiological findings specific for pulmonary nodules. For this Sub-event, attributes were established according to Lung-RADS [34], i.e. clinical guidelines defined by the American College of Radiology (ACR) to manage indeterminate pulmonary nodules. Additional attributes were included to allow localizing the referenced lesion, i.e. the *Lesion coordinates* considering the patient position as reference frame.
  - the *Other Finding Identification Sub-event* (Table S4, supplementary materials, section A), to include radiological findings that cannot be categorized as pulmonary nodules. This component contributed to documenting the presence of inflammatory conditions such as densifications, emphysema, lymph nodes abnormalities or others collateral pathological conditions.
  - The *Post-processing Sub-event* (Table S5, supplementary materials, section A), to describe a specific anatomical part whose contours are defined by a DICOM-SEG object. The attributes of this Sub-event, are mapped to a structured report file (DICOM-SR).

As per the schema in Fig. 2, an *Image Acquisition Event*, can be an effect of two different *Implications* represented by the following causal *Events,* respectively: the *Recruitment Event*, which holds information related to the eligibility of the Sample Donor into the COSMOS study (Table S11, supplementary materials, section A), or an *Imaging Analysis Event*, defined above. When connected only to the *Recruitment Event*, an *Image Acquisition Event* is simply due to the adopted protocol (baseline LDCT exam or routine annual follow-up) in case of no suspected clinical findings in an earlier *Imaging Analysis Event*. Conversely, due to the protocol rules that suspected clinical findings lead to additional medical imaging investigations (monitoring follow-up), in this case an *Imaging Analysis Event* is the causal event that leads via an *Implication* to an *Image Acquisition Event*.

A suspected clinical finding in an *Imaging Analysis Event* can also form the causal event of an *Implication* that leads to a *Biological Sampling Event* with resulting *Biological Sample*. The particulars of the *Biological Sampling Event*, includes attributes related to the invasive procedure applied to collect the biological sample (Table S7, supplementary materials, section A), whilst the *Biological Sample Analysis Event,* contains the *Pathological result* derived from the sample (Table S8, supplementary materials). Currently, the COSMOS dataset does not include digital biological imaging samples as DICOM pathology image, but if these would have been available, the *Biological Sampling Event* and *Biological Sample Analysis Event* would be replaced or integrated with an *Image Acquisition Event* and an *Imaging Analysis Event* respectively, where the latter would store the *Pathological result.*

In addition to the *Recruitment Event*, two *Events* were defined that are directly connected to the *Sample Donor,* yet independent from specific sample collections:

- The *Patient History Event* (Table S10, supplementary materials, section A), which involves information related to *Sample Donor*’s life-style (e.g. smoking exposure), symptoms (e.g. respiratory disorders) as well as pre-existing or past-pathologies (e.g. past oncologic pathologies); since many of these factors were considered among the eligibility criteria of the COSMOS study, we considered the *Recruitment Event* as an Implication of the *Patient History Event.*
- The *Diagnosis Event*, which aims at integrating findings derived from the *Imaging Analysis Event* with those of the *Biological Sample Analysis Event* to reach a unified diagnosis; indeed, in our case study, evidence derived from a digital radiological imaging can be confirmed through the analysis of a biological sample. In case of malignant lesion diagnosis, the *Grade* of the disease was documented and considered as a *Diagnosis Event* attribute (Table S12, supplementary materials, section A).

In supplementary materials, a full example of the possible clinical workflow (Figures S1 and S2, section B) in the lung cancer screening context is reported to clearly demonstrate some of relationships described in this section and to support a step-by-step evaluation of the structured database.

## Results

We have created a structured database in which specific and unambiguous queries are feasible and should allow for a comprehensive description of the COSMOS repository to be exploited for research in image processing and analysis, including radiomics and artificial intelligence, and generally, in fostering research on lung cancer management. In this section, we provide an overview of the data documented in the COSMOS structured database, considering both the biological sample and the radiological imaging sample collection.

### Imaging Sample Collection and Patient History Event

*DICOM Series Samples* in the COSMOS cohort were directly associated with two main events: an acquisition (*Image Acquisition Event*) and an analysis (*Imaging Analysis Event*) event.

**Fig. 3:**
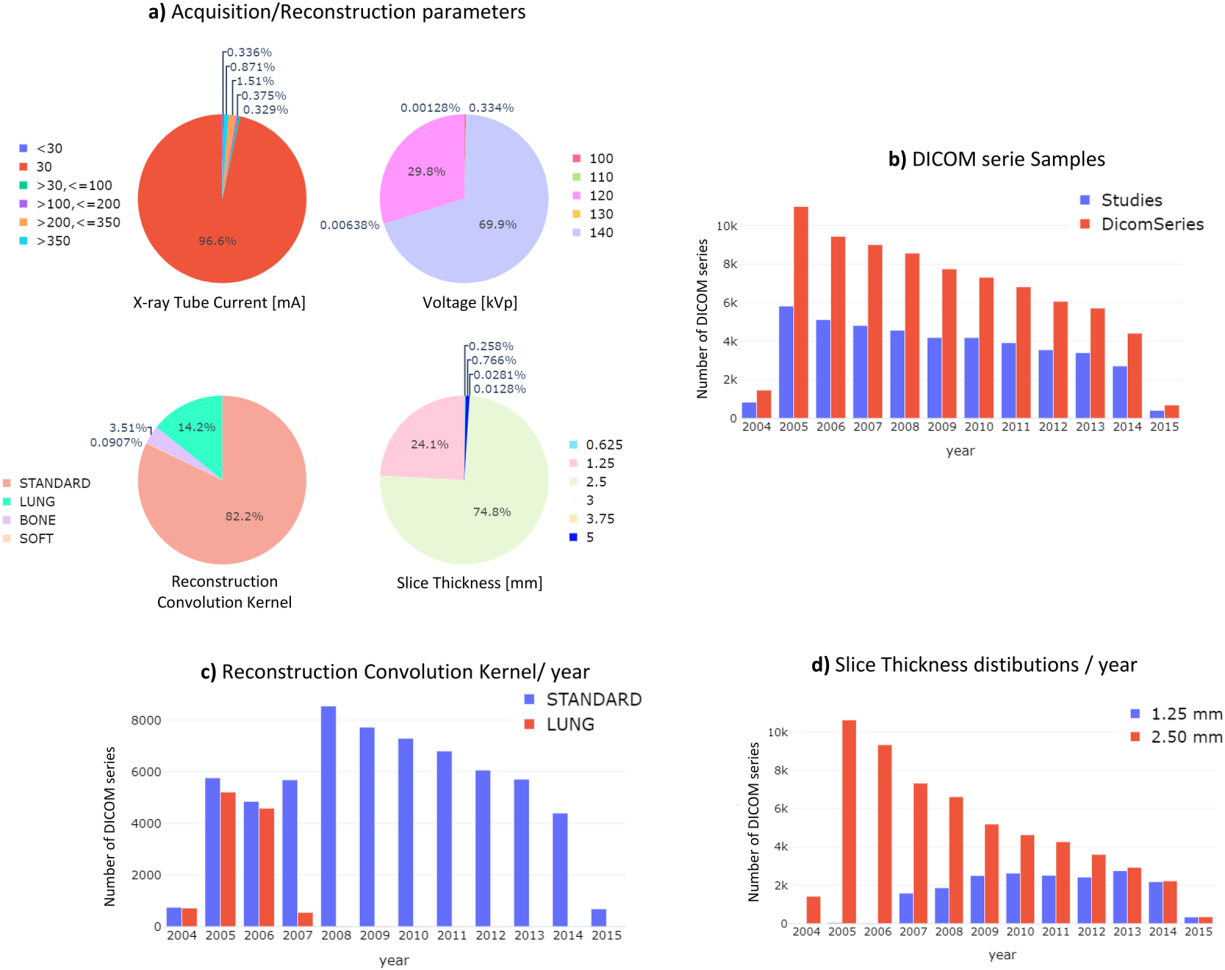
Distribution of acquisition and reconstruction parameters among the LDCT DICOM series in the COSMOS dataset. Panel (**a**) shows distributions of *X-ray* Tube current and voltage, *Reconstruction Convolution Kernel and Slice Thickness* for the entire set of LDCT scan collected during the ten years of study. The number of studies versus the number of reconstructed LDCT series; the number of series for standard and lung reconstruction kernels, and the used slice thickness (“2.5” versus “1.25” mm), by the year of the study, are shown in panels **b**, **c**, and **d**, respectively

All the *DICOM Series Samples* consisted of LDCT scans, with the most frequent combination of acquisition parameters being a 30 mA *X-ray tube current* and 120 or 140 *kVp of voltage* (Fig. 3a) [35]. From 43,000 patient studies, 73,000 DICOM series (scans) were found (Fig. 3b) due to reconstructions with both standard and lung *Convolution kernels* being performed in the early years of the COSMOS study (Fig. 3c). In the later years of the study, only the standard kernel was used. Similarly, during the first year of the study, all reconstructions were performed with 2.5 mm *slice thickness*, whereas LDCT with a reduced slice thickness (1.25 mm) were also reconstructed from year 2007 (Fig. 3d).

### Events Connected Directly and Indirectly to the DICOM Series Sample

Of the main events connected to the *DICOM Series Sample*, the *Image Acquisition Event* aims to contextualize the screening exam via an *Exam Type* attribute defined to express the role of each exam, distinguishing baseline exams (i.e. first LDCT scan acquisition of the longitudinal series) from routine or monitoring follow-ups. Specifically, routine follow-ups correspond to the annual acquisitions foreseen by the prevention program in case of absence of suspicious lesions, whereas monitoring follow-ups correspond to auxiliary acquisitions scheduled 3–6 months after a baseline or a routine follow-up where an abnormality was noted. Some 1463 of the 5206 patients underwent one or more monitoring follow-up. Thus, this *Exam Type* represents 5.3% of the DICOM studies, whereas 82.4% were routine follow-ups.

As already mentioned, the *Image Acquisition Event* can be an effect of a *Recruitment Event*. This depends itself on the *Patient History Events* component, where several habits and medical history factors that can influence the occurrence of lung cancer were included. To distinguish different risk factors, the *Risk Type* attribute was included (Table S6, Supplementary Materials, section A). Specifically, *respiratory disorders* and *oncologic history* were included as *Risk Type*s related to the patient’s medical history, while *smoking history* and *chemical exposure* were included as external risk factors. Particular attention was given to *smoking history,* which was the main eligibility criteria of the COSMOS study along with patient age. The mean (std) of the *Duration* of the smoking exposure attribute was 37.9 (6.1) and 39.9 (6.4) years for women and men, respectively. As an *Exposure Entity* attribute, the number of cigarettes smoked per day was of 24.9 (10.29).

The second main event directly connected to the *DICOM Series Sample* is the *Imaging Analysis Event* which includes the products derived from the imaging samples grouped into the following three *Sub-events*: *Pulmonary Nodule Identification, Post-processing Sub-event* and *Other Finding Identification Sub-event.*

The *Pulmonary Nodule Identification Sub-event* was dedicated to findings annotated by radiologists according to the Lung-RADS guidelines. Among its main attributes, lesion *Type – texture related* (solid, part-solid and non-solid) as well as the lesion *Diameter* are defined. To document the nodule location, image number and the lung lobe recorded during the COSMOS study were documented as *Lobe* attribute. Further attributes related to lesion localization (*Lesion coordinates*, *Type – Location related*) have been included only for a subset COSMOS so far, as the integration of this information is on-going.

**Fig. 4:**
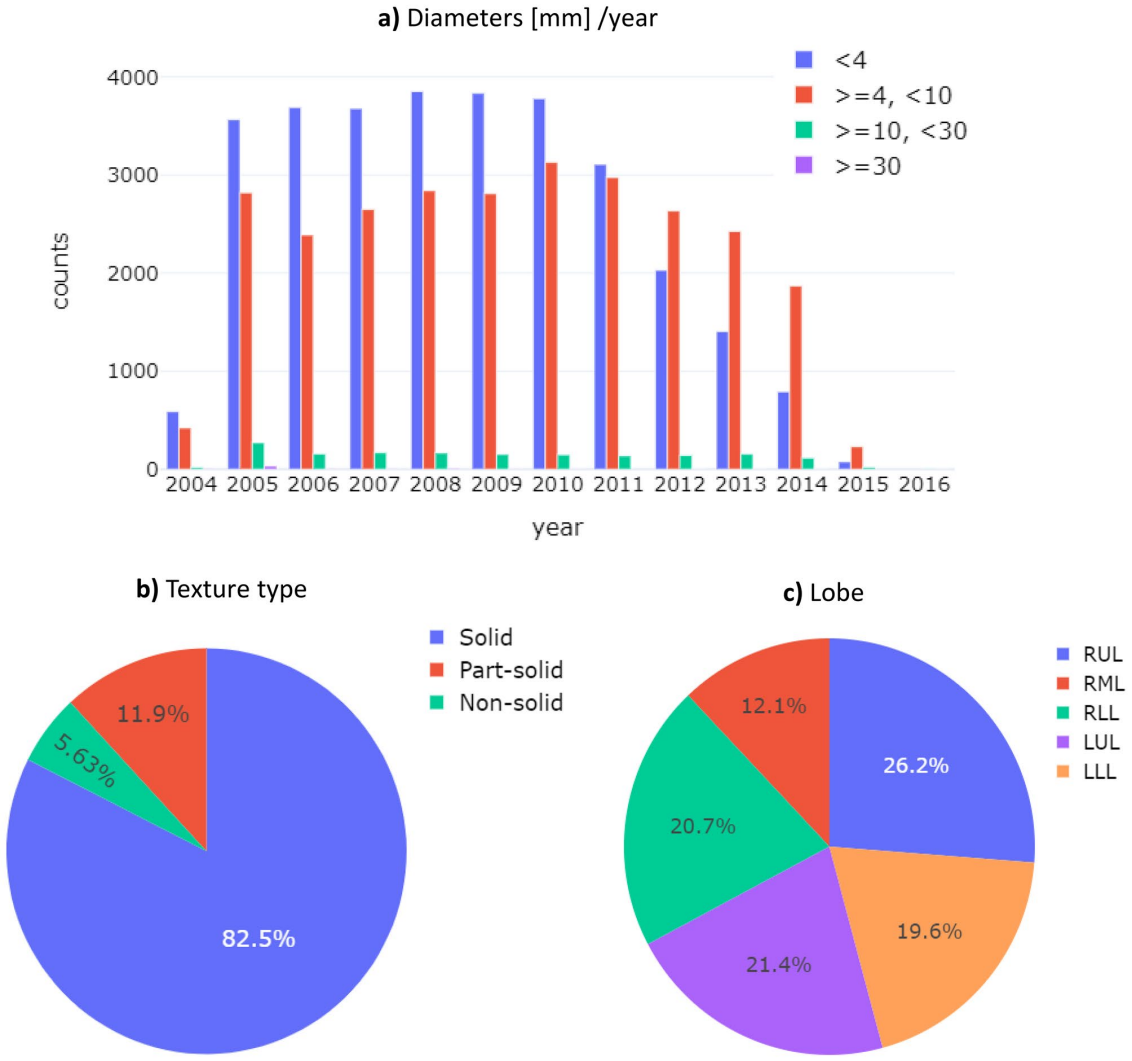
Summary of the main radiologic features regarding nodule characteristics annotated in the *Pulmonary Nodule Identification Sub-event*. On panel (**a**), the number of documented nodules over the course of the ten years of the COSMOS study is reported, subdividing them in four groups according to *Diameter* size (mm). Panel (**b**) shows lesion distribution according to *Type – texture related* attribute, whereas panel (**c**) lesion distribution according to *Lobe* attribute (RUL = Right Upper Lung, RML = Right Middle Lung, RLL = Right Low Lung, LUL = Left Upper Lung, LLL = Left Low Lung)

Among the 5206 *Sample Donors* involved in the study, those associated with *a Pulmonary Nodule identification Sub-event* gave rise to a set of 15,879 lesions, which appear in multiple exams. The distribution of nodules sizes according to *Diameter* attribute (Fig. 4a) showed most of the lesions (50.7%) to be below 4 mm, followed by 45.3% of lesions in the range 4–10 mm. As recorded in the *Type – texture related* attribute, solid nodules were more frequent (82.5%) than part-solid (11.9%) or non-solid nodules (5.6%), in agreement with published reports that solid lesions tend to be more frequent (Fig. 4b). The distribution of nodules between the lobes of the lungs (*Lobe* attribute), was relatively homogeneous (Fig. 4c), with the superior part of the right lobe being slightly predominant with respect to other locations.

The *Post-processing Sub-event* holds information derived from a DICOM-SEG object which defines the mask of a specific pulmonary nodule; therefore, it coexists with *Pulmonary Nodules Identification Sub-event*. The *ROI Generation Algorithm* within this component allows documenting whether segmentations were performed through manual or automatic contouring. The lesion *Volume*, present among Lung-RADS features, was included as attribute of the *Post-processing Sub-event*. Table 1 shows the main characteristics (*Volume, ROI Generation Algorithm* and numerosity) of the collected lesions’ contours subdivided by *Type – texture related* and *Lobe* attributes. Of 2008 lesions contoured to date, 472 were manually segmented, whereas 2747 were collected through a semi-automatic segmentation tool.

**Table 1:** Summary of the *Post-processing Sub-events.* For the two segmentation *Modality* (Manual and Automatic), the number of cases, as well as the mean and standard deviation, the minimum and maximum *Volumes,* are reported for the overall set of nodules as well as according to *Type-Texture* and *Lobe* attributes

	Manual *Modality*		Automatic *Modality*	
	Nodule size *Volume* [mm^3^]	Number of cases	Nodule size *Volume* [mm^3^]	Number of cases
Overall	2419 (7418) [17—82122]	472	328 (1496) [2—57975]	2747
Nodule *Type-Texture related*				
Solid	2352 (6065) [17—57975]	265	323 (1744) [2—57975]	1795
Part-solid	3467 (12,470) [34—82122]	101	259 (831) [2—12540]	585
Non-solid	1588 (2120) [60—12277]	106	464 (874) [12—7197]	367
Nodule position *Lobe*				
RUL	1872 (2811) [17—17699]	197	414 (1184) [2—15295]	765
RML	4365 (11,981) [26—57975]	28	356 (3169) [3—57975]	350
RLL	4902 (14,978) [21 -82122]	68	308 (1086) [2—17294]	537
LUL	1994 (6040) [19—40130]	120	265 (1046) [3—20795]	655
LLL	1325 (2765) [26—19915]	59	273 (700) [5—9790]	440

Finally, the *Other Finding Identification Sub-event* was defined to annotate radiological findings that are not pulmonary nodules and hence not covered by the *Pulmonary Nodule Identification Sub-event*. Specifically, the possible documented findings are: emphysema, lymphadenopathy, apical scar, pleural thickening, pneumothorax and pneumonia. Findings related to lesions localized in proximity of the lungs, e.g. thymus, were also recorded as *Other Finding Identification Sub-event.*

### Biological Sample Collection and Diagnosis Event

**Fig. 5:**
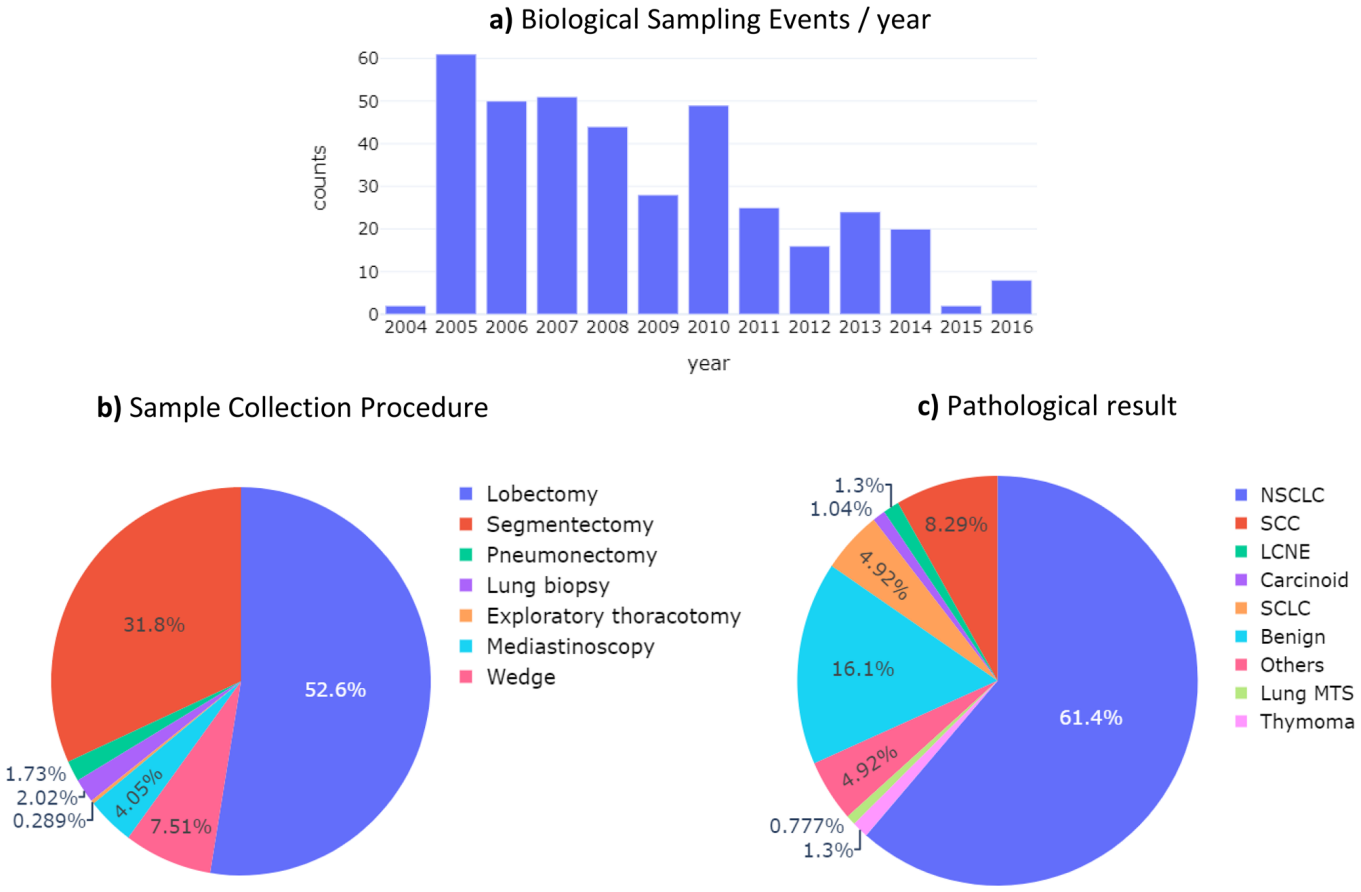
The distributions of **a**) *Biological Sampling Events* by year. **b**) *sample collection procedure* attribute, included in the Biological Sample Event (Table S2), and **c**) the *Pathological result* attribute of the *Biological Sample Analysis Event* (Table S8) for the subjects over the course of the COSMOS study

In the current repository, events connected to biological samples are available only, as particulars of the biological samples themselves or digital data through DICOM pathology samples have not yet been included in the COSMOS repository. Considering the *Biological Sampling Events,* 391 samples were recorded across 350 subjects (*Sample Donors*) with some having undergone two (33 subjects) or more (4 subjects) invasive procedures. As can be noted from Fig. 5a, *Biological Sampling Event* was more common during the first five years of the COSMOS study (2005–2010). The *Collection procedure* attribute was available for 380 samples (Fig. 5b). For almost all samples (386), the *Pathological result* attribute was known. As can be seen in Fig. 5c, 16% of the analysed samples were associated to a benign lesion.

Among the 386 *Diagnosis Events* generated as effect of the *Biological Sample Analysis Event* and from the *Imaging Analysis Event*, 300 were associated with lung lesions considered as pulmonary nodules (*Pulmonary Nodule Identification Sub-event*) and are therefore associated to radiological features according to Lung-RADS standards. Fifty-six cases were instead associated to inflammatory status of the lung or other abnormal conditions (*Other Finding Identification Event*). Because of a lack of reported radiological characteristics the remaining result derived just from the *Biological Sample Analysis Event*.

## Discussions

The standardization of information, well established for biobank data sharing, is becoming an important consideration in the field of medical imaging, driven by its role in precision medicine and CAD applications. The main aim of MIABIS lays in the definition of a standard data structure, which can facilitate both data collection and sharing. Although MIABIS has mainly referred to biological samples data, in their recent publication Eklund and colleagues (2020) stated their intent to extend the data model to biological imaging samples. In preparation for the creation of a public repository of longitudinal observations LDCT scans acquired during the COSMOS study, we have proposed an extension to MIABIS and demonstrated its use in defining a standard database structure in which to include radiological imaging data along with biological information.

Starting from the last published version of the MIABIS data model, we have proposed a series of changes to improve its generalizability. For this purpose, the list of attributes needed to define a *Sample* component was limited enlarging its field of inclusion to non-digital biological samples and digital imaging samples of a different nature (e.g. biological imaging samples and/or radiological imaging samples). While the proposed data model keeps *Sample Donor*, *Sample* and *Event* as the three main components of a data collection, we introduced the need of having multiple events with the possibility to define intra-collection links as well as inter-connections between heterogeneous *Events*. Specifically, we defined an *Implication* component, which establishes the role of one event in relation to another event (i.e. whether the event is a cause or effect of the implication). In our case-study, such relationships can be found within the *Diagnosis Event* where non-digital pathological and digital radiological outcomes are integrated. We believe the inclusion of such relationships strengthens the usability and interpretability of the repository. Indeed, among the most common aims of artificial intelligence applications, there is the prediction, from non-invasive diagnostic imaging, of target outcomes that are typically obtained through invasive procedures.

We have also proposed the possibility to have child components, i.e. *Sub-events*, of Events. For example, according to the presented case-study, the *Imaging Analysis Event* was associated with the *Pulmonary Nodule Identification Sub-event*, the *Post-processing Sub-event* and the *Other Finding Identification Sub-event* components, that are different products derived from the CT image. The inclusion of multiple Sub-events components linked to a parent *Event* makes the inclusion of lesions contours or other type of annotations more tractable. As such, the main advantage of the presented data model can be addressed to the consequent simplification of data retrieve procedure despite the large number of queryable attributes.

We used the COSMOS study as a case study consisting of patient history, LDCT imaging scans acquired over ten consecutive years, non-digital pathology results and a substantial set of labelled data that is in the process of preparation. As suggested by Eklund (2020), already existent well-defined standards were considered when available. Specifically, a subset of standard DICOM fields was adopted as attributes associated to radiological LDCT imaging samples. In this regard, the compatibility of the general data model to other imaging standards (e.g. the Brain Imaging Data Structure, BIDS, increasingly used in the field of neuroimaging experiments) needs to be evaluated in the future. Currently, digital biological samples have not yet been collected in the COSMOS study which just include *Biological Sample Analysis events*; if and when digital biological data in the form of DICOM pathology images will be available, these can be treated as non-invasive radiological DICOM images and thus considered as *Imaging Acquisition Event*. We do not expect difficulties with attributes definition as differences in DICOM tags are present also between different imaging modalities (e.g. Computerized Tomography vs. Positron Emission Tomography).

As part of the COSMOS study, pulmonary nodules were documented referring to Lung-RADS [34], which aimed to standardize the framework of lung cancer screening CT data management. Therefore, all radiological features mentioned in the Lung-RADS were included as queryable attributes in the *Pulmonary Nodule Identification Sub-event* (Table S3), with exception of *Volume,* which was included as an attribute of the *Post-processing Sub-event* being dependent on the contoured region defined by the DICOM-SEG object. *Lesion coordinates* were also included among the fundamental attributes of the *Pulmonary Nodule Identification Sub-event* to account for lesion description as pointed out also by Kostopoulos et al. (2017). As mentioned in the method section, the DICOM-SEG object was linked to a DICOM-SR with the aim of defining a structured repository where additional products related to the segmentation object can be collected, as products of radiomics-based or AI studies [31, 32]. Regarding DICOM-SEG object, DICOM RT-struct files which describe a region of interest as a list of points can be considered as an alternative way to encode segmentations despite it is considered specific to the radiotherapy field.

Comparing our data model with the information reported by Clark et al. (2009) for the NLST data collection, a larger set of queryable attributes is now available for the COSMOS data model (Supplementary Material, section D), and across a wider span of the data (imaging parameters, implications, pathological findings, lesion locations etc.). A further extension is foreseen for the COSMOS repository, consisting in the inclusion of PET-CT scans acquired according to the protocol reported in the dedicated section “The COSMOS Study Protocol”. Additionally, to allow database sharing, the deidentification process will be repeated to reach an anonymous state of the database which satisfies the GDPR definition. The radiological and biological findings associated to the COSMOS dataset had already been collected in a spreadsheet and database structures that were relatively straightforward to translate to the unified database in the demonstrated test case. Both the DICOM images, which required export from our hospital PACS, and the lesion contours being generated via dedicated software [30] are held in files that are referenced from the database. In other study contexts, it will likely be useful to integrate data from procedural reports and other clinical records, necessitating their extraction from hospital information systems and likely further refinement in order to be incorporated into the database. Our results suggest that such preparation is feasible and should not impinge the ability to integrate the derived data into the data model, though considerable initial effort may be required to ensure data integrity, completeness and appropriate definition of *Implications* between events. The transition to structured reporting and the storage of content, rather than document storage, will mark a step towards more efficient incorporation of clinical data. The growing list of imaging reporting standards for multiple anatomical sites, such as those promoted by the American College of Radiology (BI-RADS for breast cancer [36], PI-RADS for prostate cancer [37] and so on), are well-suited for incorporation into the proposed data model, and should allow easy translation to planned medical image-based studies in precision medicine. For demonstrative purposes, a second scenario related to prostate cancer diagnosis is reported in supplementary materials (section E).

## Conclusions

We proposed an extension of the MIABIS data model, previously defined to standardize biobanks description. With this work we aim to encourage the integration of different sources of data in a structured fashion driven by well-defined cause-effect relationships that reflect real clinical workflows. Adopting this structure should facilitate the research progress on precision medicine, which is often limited by the differences in database description.

As case study, the COSMOS database was considered and from the reported results an overview of the information that can be retrieved was given.

## Supplementary Information

Below is the link to the electronic supplementary material.

Supplementary file1 (DOCX 1351 KB)

## Data Availability

Data still not public available.

## Code Availability

Code still not public available.
