# Supplementary material for: Integrating Biological and Radiological Data in a Structured Repository: a Data Model Applied to the COSMOS Case Study
Source: J Digit Imaging. 2022 Mar 16;35(4):970–82. doi: 10.1007/s10278-022-00615-w (PMC9485502; doi:10.1007/s10278-022-00615-w)
Supplement: Supplementary file 1 — Supplementary file1 (DOCX 1351 KB) [file 10278_2022_615_MOESM1_ESM.docx]

# Supplementary description of the COSMOS database case study

Underlined attributes reported on the following tables represent the foreign key through which a relationship among components is established. The Patient ID is reported as foreign key just for the *Patient History Event* component.

# Digital collection components

Table S1. DICOM Series Sample.

| *Attribute name* | *Allowed values* | *Attribute description* | *Constraints* |
| --- | --- | --- | --- |
| Series Instance UID | Coded string | Random and Unique ID generated by the anonymization system |  |
| Study Date | yyyy-mm-dd Thh:mm:ss | Date and Time when the DICOM serie is acquired. According to the study type, time can be neglected considering only the date. |  |
| Body Part Examined | String | Anatomical site represented in the image. For example, *abdomen*. |  |
| Modality | String | Modality of Image acquisition (e.g. CT, PET, T2W MRI, DWI MRI etc.) |  |
| Image resolution | Vector of decimals | Spatial distance between image pixels along the two/three directions | Use either Image Resolution or the combination PixelSpacing/ Slice Thickness; not both. |
| Pixel Spacing | Vector of decimals | Spatial distance between pixels in the axial place. | Use either Image Resolution or the combination PixelSpacing/ Slice Thickness; not both. |
| Slice Thickness | Decimal | Spatial distance between images planes | Use either Image Resolution or the combination PixelSpacing/ Slice Thickness; not both. |
| Number of Slices | Decimal | Number of DICOM images which constitute a DICOM serie. Images of the same serie share the same Series Instance UID |  |
| Manufacturer Name and Model | string | Company name and name of the product release. For example, GE MEDICAL SYSTEMS (Name) – LightSpeed Ultra (Model) |  |
| Additional attributes depend on modality | _ | In our case study, only LDCT scan were included as imaging samples, thus among the main additional attributes we considered *X-ray Tube current*, *kVp* and *Reconstruction Convolution kernel.* | _ |

* With exception to the “Image Resolution”, the other attributes reported in the table correspond to DICOM fields found in LDCT DICOM series.

Table S2. Image Acquisition Event.

| *Attribute name* | *Allowed values* | *Attribute description* | *Constraints* |
| --- | --- | --- | --- |
| Acquisition ID | Coded string | ID which identifies a unique *Imaging Acquisition Event.* |  |
| Series Instance UID | Coded string | Foreign key inherited by the *DICOM Serie Sample.* |  |
| Protocol/ Study | String | The name of the adopted protocol/ study if exist (ex. COSMOS protocol, PI-RADS mpMRI) |  |
| Follow-Up number | Decimal | The number of exams along a sequence such as in image-based screening programs. |  |
| Exam Type | List: base-line, routine follow-up, monitoring follow-up | Type of exam if connected to other exams. |  |
| Motivation | Free text | Reason of the examination | English language |

## Sub-Events related to the *Imaging Analysis Event*

Table S3. Pulmonary Nodule Identification Sub-event where the list of attribute names was established according to radiological features cited in the lung-RADS recommendation.

| *Attribute name* | *Allowed values* | *Attribute description* | *Constraints* |
| --- | --- | --- | --- |
| Pulmonary nodule ID | Coded string | ID which combined with the inherit *Analysis Event ID* attribute defines a unique pulmonary nodule detected in a specific image acquisition*.* | This ID should be the same when the imaging exam is repeated at multiple time points. |
| Analysis Event ID | Coded string | Foreign key inherited by the parent Event *Imaging Analysis Event.* |  |
| Type – Texture related | List: Solid, Part-solid, Non-solid | Nodule type according to the appearance of density on the image. Solid nodules are those with highest density. |  |
| Type – Location related | List: Perifissural, Endobronchial, Other | Type of nodules according to their location with respect to lungs wall. For example, a perifissural nodule is located at the distal lung wall |  |
| Lobe | List: Sup-right, Inf-right, Middle,  Sup-left, Inf-left | Lung lobe in which the lesion is located |  |
| Diameter | Decimal | Size of the lesion [mm] calculated as the mean between the maximum diameter and the perpendicular diameter. |  |
| Lesion coordinates (XYZ) | Vector of decimals | Series of three decimals that define the position of the center of the lesion expressed in millimiters and referred to the patient position. |  |

Table 4. Other Finding Identification Sub-event.

| *Attribute name* | *Allowed values* | *Attribute description* | *Constraints* |
| --- | --- | --- | --- |
| Disease ID | Coded String | ID which combined with the inherit *Analysis Event ID* attribute defines a unique radiological finding*,* i.e. a unique Lung or collateral abnormality which cannot be classified as pulmonary nodules*.* | This ID should be the same when the Imaging exam is repeated at multiple time points |
| Analysis Event ID | Coded string | Foreign key inherited by the parent Event *Imaging Analysis Event.* |  |
| Sampling Date | yyyy-mm-dd Thh:mm:ss | Date on which the sampling operation is taken. |  |
| Disease Ontology | String | - |  |
| Disease ontology code | Coded String | - |  |

Table S5. Post-processing Sub-event.

| *Attribute name* | *Allowed values* | *Attribute description* | *Constraints* |
| --- | --- | --- | --- |
| ROI Number | Coded string | ID which combined with the inherit *Analysis Event ID* attribute defines a unique *Segmentation Sub-event,* i.e. a unique instance associated with a file which consists in a list of points which describe the region of interest. |  |
| Analysis Event ID | Coded string | Foreign key inherited by the parent Event *Imaging Analysis Event.* |  |
| ROI Generation algorithm | List: Manual, Automatic | Contouring modality applied to define the region of interest. |  |
| Tool | String | Name of the segmentation tool. |  |
| Dimensionality | List: 2D, 3D | Dimensionality of the region described by the list of points. |  |
| Area/Volume | Decimal | Area/Volume measured in mm2/mm3 according to 2D/3D Dimensionality. |  |
| Bounding box size | Vector of decimals | Series of two/three decimals (according to Dimensionality) describing the dimension of the box holding the contoured region along the two/three directions. |  |

# Biological collection components

Table S6. Biological Sample.

| *Attribute name* | *Allowed values* | *Attribute description* | *Constraints* |
| --- | --- | --- | --- |
| Biological Sample ID | Coded String | Unique ID of the sample within a  sample collection, often represented  by the sample barcode; text identifier. Sample ID meant for sharing |  |
| Detailed sample type | string | String which describes if the sample is a fluid, a tissue or something else. Examples: cancer cell lines, whole blood, plasma. |  |
| Sample creation date | yyyy-mm-dd Thh:mm:ss | Date in which the sampling operation is taken |  |
| Anatomical site ontology | string | Name of ontology used for describing the anatomical source of the sample material. |  |

** The attribute “Sample content diagnosis” was removed from the list of Biological Sample attributes and substituted by the attribute “Pathological result” on the *“Biological Sample Analysis Event”* component.

Table S7. Biological Sampling Event.

| *Attribute name* | *Allowed values* | *Attribute description* | *Constraints* |
| --- | --- | --- | --- |
| Sampling Event ID | Coded String | ID which combined with the inherit *Biological Causal Event ID* identifies a unique *Biological Sampling Event.* |  |
| Biological Sample ID | Coded String | Foreign key inherited by the *Biological Sample.* |  |
| Sampling Date | yyyy-mm-dd Thh:mm:ss | Date in which the sampling operation was performed. |  |
| Sampling Procedure | List: surgical procedure, biopsy, lobectomy, etc | The procedure that was used to extract the sample |  |

Table S8. Biological Sample Analysis Event.

| *Attribute name* | *Allowed values* | *Attribute description* | *Constraints* |
| --- | --- | --- | --- |
| Pathological Analysis ID | Coded String | ID which identifies the pathological analysis. |  |
| Biological Sample ID | Coded String | Foreign key inherited by the *Biological Sample.* |  |
| Analysis Date | yyyy-mm-dd Thh:mm:ss | Date in which the sample was analysed. |  |
| Pathological result | String / List | Text describing the pathological result inside the sample. Example: squamous cell carcinoma |  |
|  |  |  |  |

# Sample Donor information and Events directly connected to him

Table S9. Sample Donor.

| *Attribute name* | *Allowed values* | *Attribute description* | *Constraints* |
| --- | --- | --- | --- |
| Patient ID | Coded string | Unique ID code of the sample donor within the sample collection. |  |
| Sex | List: Male, Female | Biological sex of the sample donor |  |
| Birth Date | yyyy-mm-dd | Birth date of the sample donor |  |
| Related collection | List: Biological Sample Collection, Imaging Sample collection | List of sample collections in which the donor is involved |  |

Table S10. Patient History Event.

| *Attribute name* | *Allowed values* | *Attribute description* | *Constraints* |
| --- | --- | --- | --- |
| Risk factor ID | Coded string | ID which combined with the inherit *Imaging Causal Event ID* identifies a unique *Risk factors and Patient History Event.* | It can be the same for more than one Imaging sample since a specific risk can bring to the acquisition of multiple exams. |
| Patient ID | Coded String | Foreign key inherited by the *Sample Donor.* |  |
| Risk type | List: Smoking exposure, Urinary difficulties, Chemical exposure, Respiratory Disorders, Respiratory Pathologies, Oncologic Pathologies, Cardiological Pathologies, Others | The type of risk connected to the digital sample. |  |
| Collection date | yyyy-mm-dd | Date on which Patient information were collected. |  |
| Start Date | yyyy-mm-dd | The date on which the exposure started or at which the past pathology was diagnosed. It could be also partial, i.e. only the year can be reported. |  |
| Stop Date | yyyy-mm-dd | The date on which the exposure stopped or at which the past pathology was considered as fixed. | It can be missed if Duration is reported. |
| Duration | Decimal | Duration of the exposure/pathology expressed in Number of years. If the pathology/exposure lasts less than 1 year, the duration will be a number less than 1. | It should be reported if the exposure/pathology is still present. It can be reported as alternative to Stop Date. |
| Exposure entity | Decimal, grade, others | Quantitative documentation of the level of exposure whose metric will depend on the *Risk Type* attribute. |  |

Table S11. Recruitment Event.

| *Attribute name* | *Allowed values* | *Attribute description* | *Constraints* |
| --- | --- | --- | --- |
| Event ID | Coded string | Unique ID code which identifies the justification for the inclusion of the Sample Donor into the study. |  |
| Eligibility | List: Yes, No | Attribute which states if the Sample Donor is eligible or not into the protocol (ex. COSMOS study, PI-RADS mpMRI). |  |
| Motivation | Free text | Justification for the inclusion or exclusion from the COSMOS study | English language |

Table S12. Diagnosis Event.

| *Attribute name* | *Allowed values* | *Attribute description* | *Constraints* |
| --- | --- | --- | --- |
| Diagnosis ID | Coded String | ID which identifies a unique *Diagnosis Event.* |  |
| Grade | Coded string | Grade of the lesion according to TNM staging |  |
| Disease Ontology | String |  |  |
| Disease ontology code | Coded String |  |  |

Table S13. Implication component.

| *Attribute name* | *Allowed values* | *Attribute description* | *Constraints* |
| --- | --- | --- | --- |
| Causal ID | Coded String | ID of the *Event* which represents the cause of a second *Event,* i.e. the effect. |  |
| Effect ID | Coded string | ID of the *Event* generated as effect of a previous *Event, i.e.* the cause. |  |

# Example of the possible use of the structured database in the clinical practice

In the following example, the structured database is applied in a lung cancer screening context: an instantiation is reported in Figure S1 together with a temporal workflow (Figure S2) which describes the sequence of events. The temporal workflow is also aimed at performing a step-by-step evaluation of the proposed structured repository.

As depicted in Figure S1, the *Sample Donor* results a heavy smoker from the *History Event* (he/she smoked for more than 15 years a number of cigarettes higher than 20 packs per year); therefore, he/she is recruited into the COSMOS Lung Cancer Screening study.

The baseline acquisition (first CT scan) is therefore planned (*Image Acquisition Event* with ID IA000101): in date 2005-05-04 the first CT scan is acquired (*DICOM Serie sample* with ID UID00045675464883) and from the *Imaging Analysis Event* a suspected lung lesion was found (pulmonary nodule with ID PN000101). A follow-up at three months was therefore required to monitor the dynamic of the lesion.

A second acquisition is therefore planned (*Image Acquisition Event* with ID IA000102): in date 2005-08-04 the second CT scan is acquired (*DICOM Serie sample* with ID UID00045675464907) and from the *Imaging Analysis Event* suspected pulmonary nodules results larger (Diameter from 8.3 to 15.5 mm).
A further investigation is therefore needed: a biopsy (Biological Sample Event with ID BSE000101) is planned. The outcome of the pathological analysis results in a squamous cell carcinoma.
Integrating the outcomes of the *Biological Sample Analysis Event* (ID BSA000101) and the *Imaging Analysis Event* (ID CTA000102), the final diagnosis is that the *Sample Donor* is affected by a tumour at stage IA according to TNM classification.


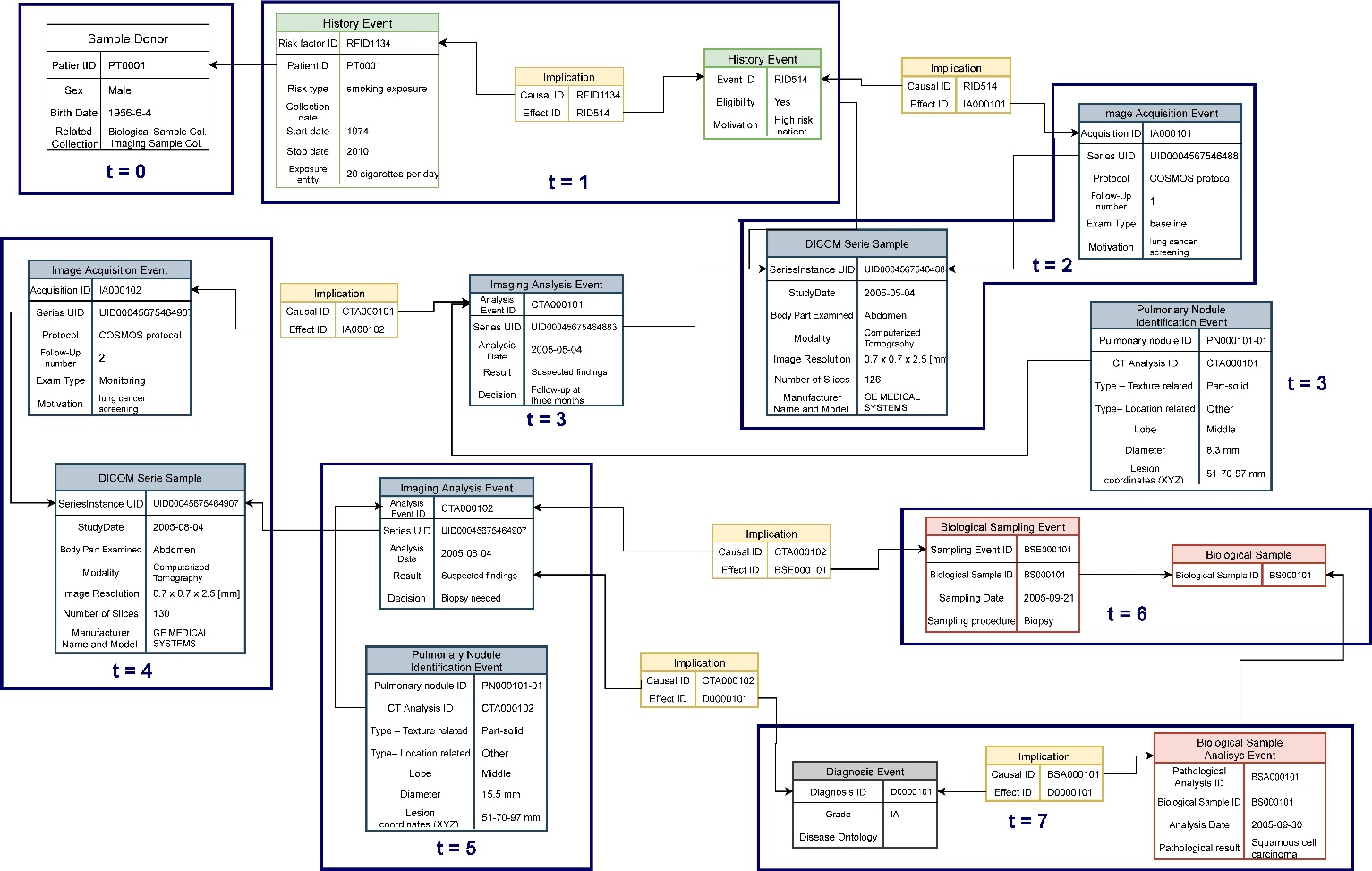


**Figure S1.** Example of the possible use of the structured database in the clinical practice. The direction of the arrows indicates where the common attribute comes from: the arrow head point to the component for which the attribute is a primary key while the tail refers to the component that has inherited the attribute.


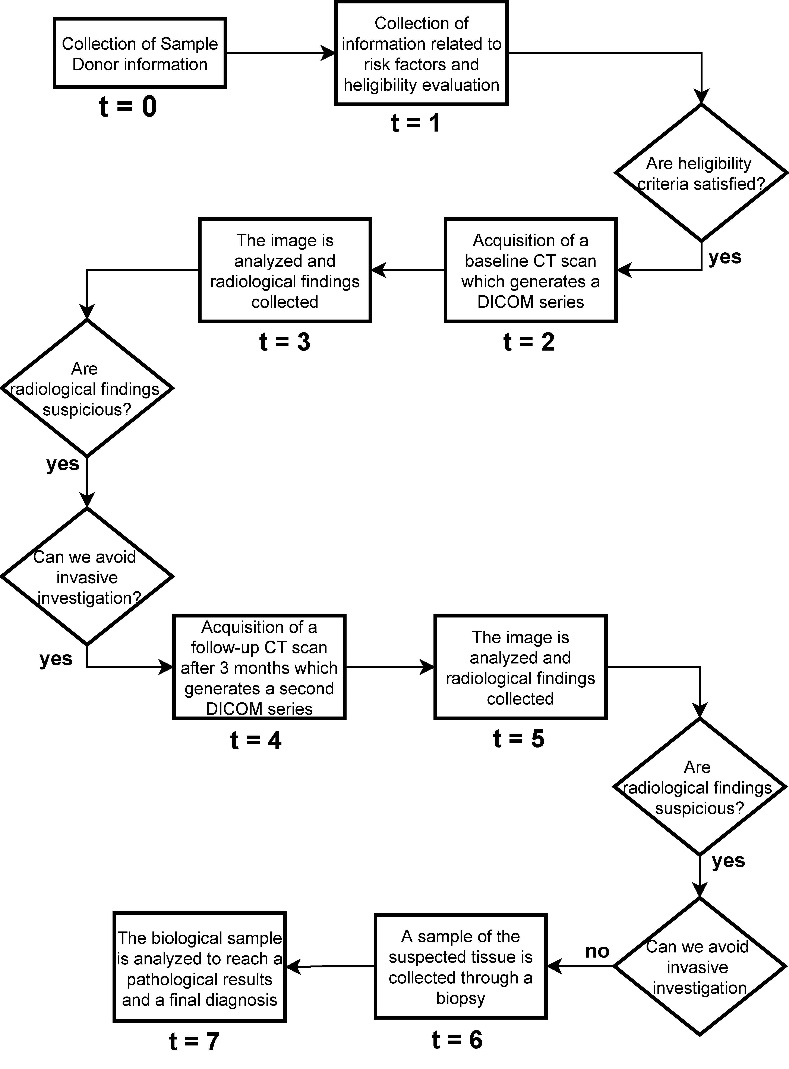


**Figure S2.** The flowchart shows the temporal sequence of the events which bring to the population of the database.

# Meaning of Entity-Relationship connection symbols


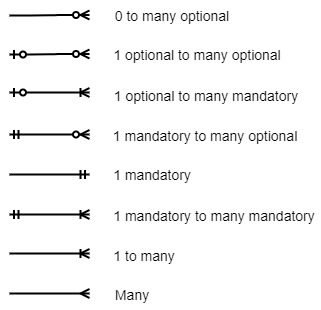


## Querying examples for data retrieval

Query example 1

Extract the list of pulmonary nodules identified at more than one time point. Consider only the time points for which we dispose of a segmentation event.

SELECT PatientID,PolmonaryNoduleID, COUNT(*) FROM

(SELECT * FROM PulmonaryNoduleIdentificationTable INNER JOIN SegmentationTable

ON PulmonaryNoduleIdentificationTable.AnalisysID = SegmentationTable.AnalisysID) AS childTableA

GROUP BY PatientID, PolmonaryNoduleID

HAVING COUNT(*) > 1

Query example 2

Extract the list of pulmonary nodules marked as adenocarcinoma (‘ADK’ as pathological result).

SELECT * FROM (

SELECT PatientID, PolmonaryNoduleID,DiagnosisID FROM PulmonaryNoduleIdentificationTable INNER JOIN

(SELECT * FROM ImageAnalisysTable INNER JOIN

(SELECT * FROM ImageAnalisysTable INNER JOIN ImplicationTable

ON ImageAnalisysTable.AnalisysID = Implication.CausalID) AS childTableA

ON childTableA.EffectID = DiagnosisTable.DiagnosisID ) AS childTableB

ON PulmonaryNoduleIdentificationTable.AnalisysID = childTableB.AnalisysID) AS childTableC

INNER JOIN

(SELECT DiagnosisID,PathologicalResult FROM BiologicalSampleAnalisysTable INNER JOIN

(SELECT * FROM BiologicalSampleAnalisysTable INNER JOIN ImplicationTable

ON BiologicalSampleTable.AnalisysID = Implication.CausalID) AS childTableD

ON childTableD.EffectID = DiagnosisTable.DiagnosisID ) AS childTableE

ON childTableC.DiagnosisID = childTableE.DiagnosisID

WHERE childTableE.PathologicalResult = 'ADK'

Query example 3

Extract the list of DICOM series acquired with specific acquisition parameters (kVp, X-ray Tube current, Reconstruction convolution kernel equal to 120, 30 and 'STANDARD' respectively) and where at least a pulmonary nodule was identified.

SELECT * FROM DICOMseriesTable INNER JOIN (

SELECT SeriesInstanceUID FROM ImageAcquisitionTable INNER JOIN

(SELECT * FROM PulmonaryNoduleIdentificationTable INNER JOIN ImplicationTable

ON PulmonaryNoduleIdentificationTable.AnalisysID = Implication.EffectID) AS childTableA

ON ImageAcquisitionTable.ImageAcquisitionID = childTableA.CausalID) AS childTableB

ON DICOMseriesTable.SeriesInstanceUID = childTableB.SeriesInstanceUID

WHERE DICOMseriesTable.kVp = 120 AND DICOMseriesTable.XrayTubeCurrent = 30 AND

DICOMseriesTable.ReconstructionConvolutionkernel = 'STANDARD'

# Additional example of the possible use of the structured database in the clinical practice of prostate cancer diagnosis

In the following example, the structured database is applied according to the PI-RADS standards adopted for prostate cancer diagnosis. An instantiation is reported in Figure S3 together with a temporal workflow (Figure S4) which describes the sequence of events. The temporal workflow is also aimed at performing a step-by-step evaluation of the proposed structured repository.

From Figure S3, the *Sample Donor* results a subject affected by urinary problems in the *History Event;* therefore, he can be considered at risk of prostate cancer occurrence.

The first clinical investigation consists in the acquisition of a blood sample (*Biological Sampling Event* with ID BSE000101) to measure the prostate-specific antigen (PSA) level (*Biological Sample Analysis Event* with ID BSA000101). PSA level above the threshold implicates the acquisition (*Image Acquisition Event* with ID IA000101) of a multi-parametric Magnetic Resonance Imaging (mpMRI) as suggested from the PI-RADS guidelines. As result from the latter event, two DICOM series are derived: a T2 weighted (T2w) MRI (*DICOM Serie Sample* with ID UID00045675464907) and a diffusion weighted imaging (DWI) MRI (*DICOM Serie Sample* with ID UID00045675464908). From the analysis of the two images (with MRIA000101 and MRIA000102 IDs of the analysis), a prostate lesion with ID PL000101 is identified and described through attributes derived from the T2W MRI (Volume, coordinates) and from the DWI (ADC value) reaching a risk score equal to 3 from both exams. With an average score equal to 3, a dynamic contrast enhanced MRI (DCE MRI) needs to be acquired (*Image Acquisition Event* with ID IA000102 from which the *DICOM Serie Sample* with ID UID00045675464909 is obtained) according to PI-RADS protocol. From the analysis (ID equal to MRIA000103) of the DCE MRI the presence of the suspicious lesion is confirmed (*Prostate Abnormality Identification Event* with ID PL000101) due to the presence of pathologic enhancement. This implicates the acquisition of a tissue sample (Biological Sample Event with ID BSE000102) which was digitalized as DICOM pathology image (UID00045675464910). From the analysis (*Imaging Sample Analysis Event* ID PIA000101) of the digitalized biological sample combined with mpMRI results, the final diagnosis (*Diagnosis Event* identified by ID D000101)) of prostate cancer is reached.

With respect to the application within the COSMOS dataset, the only different attributes for the application to a prostate diagnosis case are reported in Table S14 and S15.


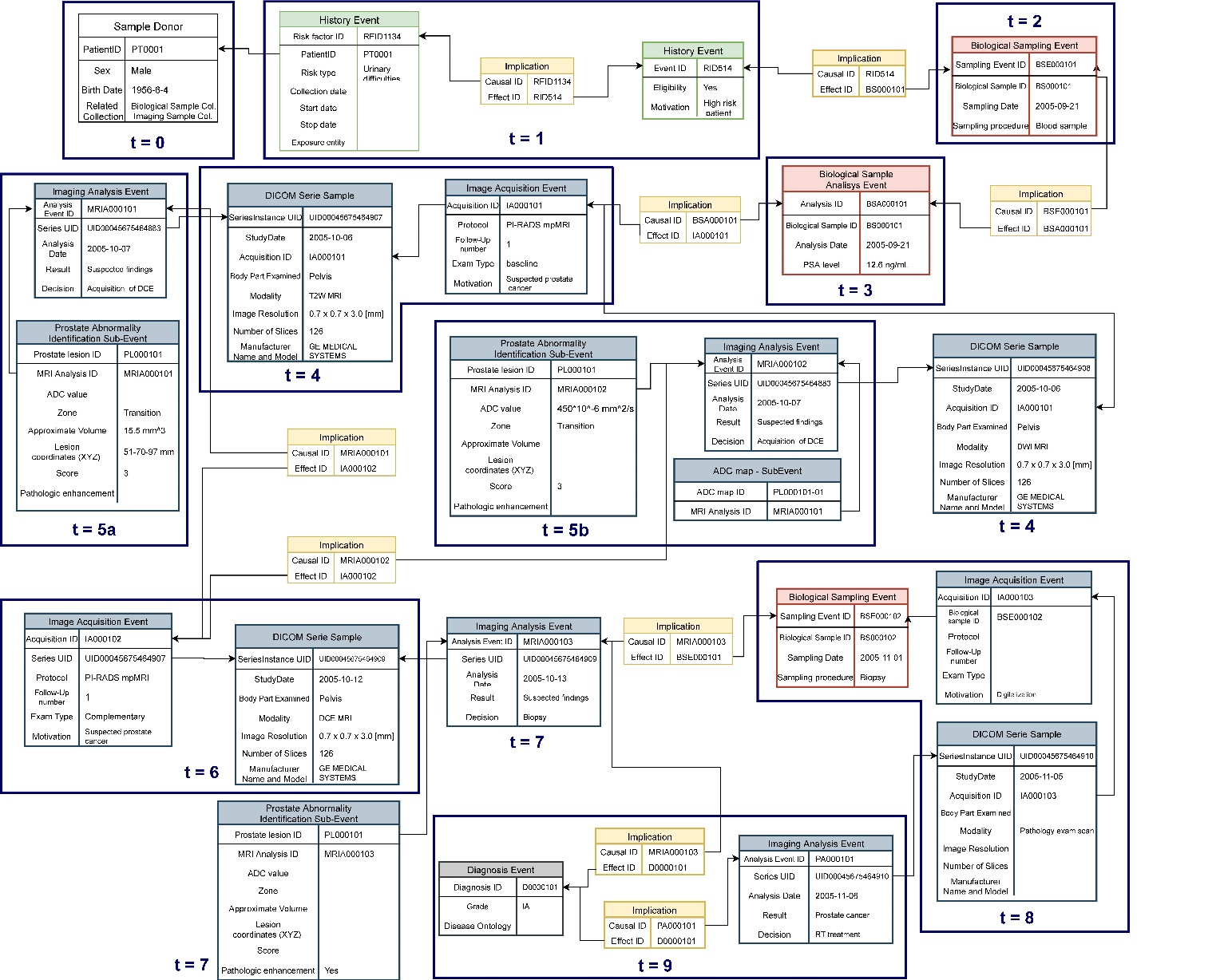


**Figure S3.** Example of the possible use of the structured database in the clinical practice into a context of prostate diagnosis. The direction of the arrows indicates where the common attribute comes from: the arrow head point to the component for which the attribute is a primary key while the tail refers to the component that has inherited the attribute.


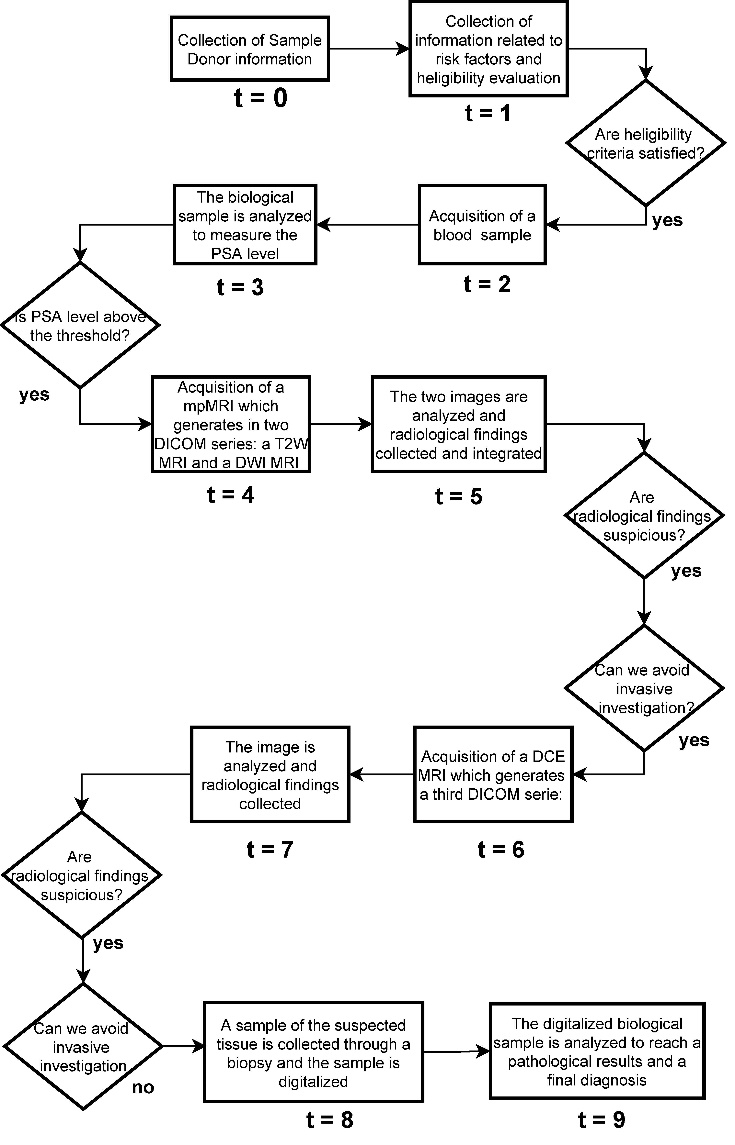


**Figure S4.** The flowchart shows the temporal sequence of the events which bring to the population of the database.

Table S14. Prostate abnormality Identification Sub-event where the list of attribute names was established according to radiological features cited in the PI-RADS recommendation.

| *Attribute name* | *Allowed values* | *Attribute description* | *Constraints* |
| --- | --- | --- | --- |
| Prostate lesion ID | Coded string | ID which combined with the inherit *Analysis Event ID* attribute defines a unique prostate lesion identified through the combination of MRI acquisitions (T2W MRI, DWI MRI, DCE MRI)*.* | This ID should be the same when the imaging exam is repeated at multiple time points. |
| Analysis Event ID | Coded string | Foreign key inherited by the parent Event *Imaging Analysis Event.* |  |
| ADC value | Decimal | Minimum or mean value found in the ADC map (Sub-Event derived from DWI MRI) inside the region of interest. Considered as an indicator of abnormality of the tissue which usually show a restricted diffusion compared to normal tissues. | A threshold between 750/900${\mu m}^{2}/sec$ is considered to differentiate benign from malignant lesions. |
| Zone | List: Peripheral, Transition, Central | Anatomical portion of the prostate where the lesion is located. |  |
| Approximate Volume | Decimal | Size of the lesion [mm3] calculated through the following ellipsoid formulation: (maximum AP dimension) * (maximum longitudinal dimension) [both placed on the mid-sagittal T2W image] * (maximum transverse dimension) [placed on the axial T2W image] * 0.52. |  |
| Lesion coordinates (XYZ) | Vector of decimals | Series of three decimals that define the position of the center of the lesion expressed in millimiters and referred to the patient position. |  |
| Score | Decimal | Metrics used to summarize levels of suspicion or risk. | Integer value within the range 1-5. |
| Pathologic enhancement | List: Yes, No | The presence of enhancement is established from the DCE MRI. It can help in the identification of small significant cancers or to confirm the suspicious nature of the lesion which results doubtful from T2W and DWI MRI inspection (score = 3) |  |

Table S15. ADC-map Sub-event.

| *Attribute name* | *Allowed values* | *Attribute description* | *Constraints* |
| --- | --- | --- | --- |
| ADC-map ID | Coded string | ID which combined with the inherit *Analysis Event ID* attribute defines a unique ADC-map which is derived from a DWI MRI to evaluate the diffusion ability of tissues. |  |
| Analysis Event ID | Coded string | Foreign key inherited by the parent Event *Imaging Analysis Event* which is referred to a DWI MRI*.* |  |
